# Understanding of and Barriers to Electronic Health Record Patient Portal Access in a Culturally Diverse Pediatric Population

**DOI:** 10.2196/11570

**Published:** 2019-04-26

**Authors:** Daniel J Miklin, Sameera S Vangara, Alan M Delamater, Kenneth W Goodman

**Affiliations:** 1 University of Miami Miller School of Medicine Miami, FL United States; 2 University of Miami Miller School of Medicine Mailman Center for Child Development Miami, FL United States; 3 University of Miami Miller School of Medicine Institute for Bioethics and Health Policy Miami, FL United States

**Keywords:** electronic health records, physician-patient relations, adolescent health, patient-accessible electronic health records, patient portals

## Abstract

**Background:**

Electronic health records (EHRs) have become a standard in the health care setting. In an effort to improve health literacy, foster doctor-patient communication, and ease the transition from adolescent to adult care, our institution created a policy that allows patients aged between 13 and 17 years to have EHR portal access. A literature review revealed predictable differences in portal registration among different ethnicities and socioeconomic statuses. Consequently, a cross-sectional survey was developed to investigate barriers to EHR portal access in a sample of culturally diverse adolescents.

**Objective:**

The aim of this study was to assess for barriers to EHR portal access in a culturally diverse adolescent population.

**Methods:**

A 42-item anonymous survey was completed by 97 adolescents aged between 13 and 18 years, attending general pediatrics clinics. The results were analyzed using descriptive statistics and *t* tests.

**Results:**

The average participant age was 15.5 (SD 1.5) years with 60% (58/97) male and 40% (39/97) female. Participants were 44% (43/97) black, 41% (40/97) Hispanic, 9% (9/97) Caucasian, 3% (3/97) Asian, and 2% (2/97) others. There were statistically significant differences in perceptions of confidentiality in age (13 to 15 years vs 16 to 18 years; *P*=.001) and insurance status (government vs private; *P*=.012) but not in gender, ethnicity, or parental education level. Younger adolescents with governmental insurance were more confident in the level of confidentiality with their physician. A total of 94% of participants had heard of the term *EHR*, but only 55% were familiar with its function. Furthermore, 77% of patients primarily accessed the internet through phones, and 50% of participants knew that patients aged under 18 years could obtain care for mental health, substance abuse, sexual health, and pregnancy.

**Conclusions:**

This research has identified gaps in EHR technology with regard to the pediatric patient population. The results of our survey show that adolescents may have misconceptions regarding the doctor-patient relationship, their ability to obtain care, and the modalities present in an EHR. As technology progresses, it is essential to have a deeper understanding of adolescents’ perceptions of confidentiality, technology, and available resources to design an EHR system that encourages patient education and communication while limiting barriers to care.

## Introduction

### Background

Since the passing of the Health Information Technology for Economic and Clinical Health Act within the American Recovery and Reinvestment Act of 2009, electronic health record (EHR) systems have become a standard of care within the health care field [1]. Providers are not only encouraged to use these systems but are also reimbursed for their *meaningful use*. These systems have been shown to provide benefits for patients and clinicians in terms of improved access to information, reduction of errors, faster test results, and improved outcomes [2-4]. However, EHR systems may lack some of the resources needed to address the complexities that exist in adolescent care settings with regard to confidentiality, autonomy, and access to care [3,5].

Adolescent patients, although formally considered part of the realm of pediatrics, provide an interesting challenge to health care providers because of their physical, social, and emotional changes. These patients are distinctive owing to the rapid development of autonomy, self-awareness, and responsibility. The Society for Adolescent Health and Medicine has affirmed that confidentiality is essential for the development of maturity, autonomy, and the willingness of adolescent patients to disclose sensitive information and seek care [6-8]. Special considerations are therefore needed when caring for this population, particularly when dealing with sensitive information such as sexual activity, pregnancy, substance use, and mental health. All or most of this *information* is available through patient EHR portals, although the portals lack functionality to manage confidentiality in an adequate manner. What information is present, how it is accessible, and what resources exist for learning and communicating with the physician can play a vital role in the care of an adolescent [8,9].

### Objectives

The University of Miami and Jackson Memorial Health Systems are 2 major academic medical centers in an urban setting that are frequently presented with a number of medically and ethically challenging pediatric cases. Miami-Dade County is unique in its ethnically and socially diverse community and has the highest rate of new HIV infections in the country, with up to 34% of new cases occurring between ages 13 and 29 years [10]. In Florida, like many other states, pediatric patients are legally able to consent for care that involves sexually transmitted infections (STIs), drug and alcohol abuse, pregnancy, and mental health [11]. This presents a challenge toward confidentiality when trying to document and bill for care in any of these areas. Although the patient can legally consent for these services, it is often their parent/guardian who pays for them. Furthermore, because of their limited interaction with the nonclinical aspects of health care, adolescents often lack knowledge of the resources and privileges available to them [6,12,13]. Therefore, in an effort to improve health literacy, foster communication between patients and providers, and ease the transition from adolescent to adult care, members of the Ethics Committee at Holtz Children’s Hospital sought to create a new policy for allowing pediatric patients to have proxy access to their EHR through a Web-based portal. A literature review and direct contact with 8 major academic and community hospitals around the country suggested that very few institutions have formal policies regarding pediatric patient access to their EHR portals [14-16]. On the basis of the information collected, the decision was made to create a policy allowing adolescent patients aged between 13 and 17 years to have proxy access to their EHR portal.

Studies suggest that there are predictable differences in portal registration patterns among different ethnicities and socioeconomic statuses [17-20]. These studies demonstrated a decrease in portal enrollment and activation by black and Hispanic adolescents, particularly those with governmental insurance [17]. Miami-Dade County has a very ethnically diverse population, with 18% black and 69% Hispanic residents and 20% of residents under the age of 18 years [21]. To our knowledge, no studies exist regarding pediatric patient perception and preferences for an EHR portal. Therefore, as a follow-up to our institution’s newly approved policy for proxy EHR portal access, a 42-item anonymous survey was created to investigate barriers to accessing EHR portals in this culturally distinct population.

## Methods

### Survey Creation

A 42-item survey was created to assess for barriers to and understanding of EHR portals for participants aged between 13 and 18 years. A literature review of existing surveys regarding EHR use demonstrated that there were very few published questions that would be applicable to a pediatric patient population. Survey items were therefore created de novo, including questions concerning the perception of confidentiality in the doctor-patient relationship, understanding of EHR systems, and knowledge of access to care as a pediatric patient. The completed survey was sent for review for clinical relevance by practicing pediatricians from Jackson Memorial Hospital and the University of Miami Health Systems. The University of Miami Biostatistics Collaboration and Consulting Core assessed the survey for question design and organization. Once the final survey was completed, questions were grouped together based on the content and separated into 6 sections: (1) what is a medical record and how does it work, (2) abilities when using a computer, (3) statement questions, (4) assessing knowledge of confidentiality, (5) obtaining care, and (6) understanding cultural barriers (Multimedia Appendix 1). These groupings were created to assist with the survey ease of use and eventual data analysis. Survey packets containing an informational flyer, consent forms, demographic section, and the 42-item survey were assembled and individually labeled for distribution.

The study was approved by the University of Miami Institutional Review Board (IRB; study number 20160624).

### Survey Distribution

Participants were identified by the age criteria before their arrival and were recruited in the waiting rooms at 3 different IRB-approved general pediatric clinics at both institutions. Inclusion criteria included subjects aged between 13 and 18 years, proficient in written and spoken English, and who were comfortable filling out the survey without parental assistance. Although the intended proxy portal would not be applicable to those aged 18 years and above, it was believed that the feedback from those soon to be transitioning into adult care was vital for the study. Upon arrival to the clinic, participants and their guardians were greeted and informed of the nature of the study. If both the adolescents and their parents or guardians expressed interest, written consent was obtained by the guardians or adolescents aged 18 years and written assent was obtained by all adolescents aged between 13 and 17 years. For accuracy purposes, the demographic portion of the survey was completed separately by the parent or guardian. Surveys were completed in the clinic waiting rooms. To increase validity, guardians were encouraged to allow participants to complete surveys by being apart from them, without assistance. Participants who completed the survey were given a US $5 gift card. The survey distribution was performed by IRB-approved study coordinators.

### Analysis

The survey responses were coded into IBM’s SPSS statistical software and questions were assessed for response frequency, mean, mode, and SD. To compare responses between groups of participants, 2 different subgroups were created using the mean score of select questions addressing similar themes. The first subsection, *Sum of Perceptions Regarding Confidentiality in the Doctor-Patient Relationship*, comprised the sum and mean of survey questions 21, 22, and 23. The second subsection, *Cultural Barriers*, comprised the sum and mean of questions 20, 31, 32, and 33 (Multimedia Appendix 1). These questions were chosen for analysis owing to previous literature reporting differences in communication, technology, and services rendered among different ethnicities and age groups [9,12,13,22-24]. The mean scores for these subsections were then compared using demographic parameters including ethnicity, highest level of parental education, gender, and age. The scores were compared using an independent sample *t* test.

The objectives of this study included understanding (1) perceptions of adolescents with regard to patient-physician relationship, (2) adolescents’ knowledge of EHR systems, and (3) practical concerns of designing an EHR system that best protects patient confidentiality while helping to facilitate patient-physician communication to improve health outcomes and patient adherence.

## Results

### Survey Demographics

There were 97 completed surveys (96% (97/101) of those approached) with an average participant age of 15.5 (SD 1.5) years with 60% (58/97) male and 40% (39/97) female participants. The majority of survey participants were black (44%, 43/97) and Hispanic (41%, 40/97), with substantially fewer Caucasian, Asian, and other participants. Insurance status was mainly governmental or public (48%, 46/97) or private (27%, 26/97), followed by hospital-based (5%, 5/97) or unknown (20%, 18/97). The level of parental education was stratified into 5 categories but grouped together into high school or below (46%, 45/97) or college and above (54%, 52/97). In total, 4 individuals declined to participate in the survey with an average age of 16.00 (SD 2.00) years. These data points included 1 male and 3 females, 1 private insurance and 3 governmental or public insurance, and 2 with parental education of high school or below and 2 with parental education of college or above.

### Statistical Analyses

Both descriptive statistics and *t* tests were employed in this study. For descriptive statistics, clinically relevant questions from the 42-item survey were classified under 5 categories: (1) perceptions regarding confidentiality in the doctor-patient relationship, (2) understanding of EHR systems, (3) practical concerns for the design of an EHR system, (4) cultural barriers affecting care, and (5) knowledge of access to care (Tables 1-6). Participants rated each statement on the survey on the Likert scale with the value *1* classified as *least true* and the value *10* classified as *most true*. The mean scores, SDs, and mode values for the descriptive statistics were interpreted on the Likert scale (Tables 7 and 8). Independent *t* tests were performed for the subsections (1) perceptions regarding confidentiality in the doctor-patient relationship and (2) cultural barriers (Tables 9 and 10). The *t* tests examined the patient ethnicity (black vs Hispanic), parents’ highest level of education (high school vs college and above), patient gender (male vs female), and age (13 to 15 years vs 16 to 18 years).

**Table 1:** Questions regarding confidentiality in the doctor-patient relationship.

Survey question	Mean (SD)	Mode	Frequency true (%)	Frequency false (%)
21. My doctor tells my parents about all our conversations, even those we have in private	5.794 (3.649)	10	—^a^	—
22. My doctor will tell my parents if I am smoking marijuana or drinking alcohol	4.505 (3.773)	1	—	—
23. My doctor will not tell my parents if I am having sex	5.041 (3.6)	1	—	—
26. Parents must be in the room when kids under 18 years see the doctor	—	—	34	66

^a^Denotes inapplicable data points.

**Table 2:** Questions regarding understanding of electronic medical record systems.

Survey question	Frequency true (%)	Frequency false (%)
1. I have heard the term medical record before	94	6
2. When my parents log in to the hospital website, they can read about what my doctor and I talked about during the visit	55	45
5. Patients can go online to see information about their visit to the doctor	70	30

**Table 3:** Questions regarding practical concerns for the design of an electronic medical record system.

Survey question	Mean (SD)	Mode	Frequency true (%)	Frequency false (%)
3. I would like to log in to an electronic medical record so I can see my health information	—^a^	—	84	16
16. I want my record to show what medicines I am taking	8.629 (2.205)	10	—	—
17. I want to be able to see my test results online	8.464 (2.471)	10	—	—
18. I would be comfortable sending my doctor messages online	7.155 (2.848)	10	—	—

^a^Denotes inapplicable data points.

**Table 4:** Preferences toward internet access and use.

Survey question and modality		Percent (%)
**11. I access internet mainly through (select all that apply)**		
	Phone	77
	Home computer	26
	Public library	4
	School	5
	Others	4
**12. I use the internet mainly for (select all that apply)**		
	School work	63
	Social media	50
	Email	28
	Games	44
	Others	8

**Table 5:** Questions regarding cultural barriers affecting care.

Survey question	Mean (SD)	Mode	Frequency true (%)	Frequency false (%)
20. I trust my doctor	9.33 (1.539)	10	—^a^	—
31. I would be willing to talk to my doctor about my boyfriend or girlfriend	6.67 (3.201)	10	—	—
32. I would be willing to talk to my doctor about sex	7.258 (2.91)	10	—	—
33. I would be comfortable talking to the doctor alone	8.082 (2.656)	10	—	—
25. Sometimes it’s hard for me to tell the doctor everything as I’m afraid he or she might judge me	—	—	34	66

^a^Denotes inapplicable data points.

**Table 6:** Questions regarding knowledge of access to care.

Survey question	Mean (SD)	Mode	Frequency true (%)	Frequency false (%)
24. Kids under 18 years can get screened for sexually transmitted diseases without their parents’ knowledge or consent	6.299 (3.395)	10	—^a^	—
28. Patients under 18 years can get a pregnancy test at the doctor’s office without parents’ permission	—	—	41	59
29. Patients under 18 years can get treatment for certain conditions such as depression, drug addiction, and sexually transmitted diseases without their parents’ knowledge or consent	—	—	50	50

^a^Denotes inapplicable data points.

**Table 7:** Mean response to questions regarding the perception of confidentiality in the doctor-patient relationship stratified by ethnicity.

Ethnicity	Mean value of response			Average of 3 questions
	Question 21^a^	Question 22^b^	Question 23^c^	
Caucasian	7.556	7.333	5.667	6.852
Hispanic	5.900	4.975	5.125	5.333
Black	5.349	3.535	4.721	4.535
Asian	5.667	3.00	6.333	5.000

^a^My doctor tells my parents about all our conversations, even those we have in private.

^b^My doctor will tell my parents if I am smoking marijuana or drinking alcohol.

^c^My doctor will not tell my parents if I am having sex.

**Table 8:** Mean response to questions regarding cultural barriers stratified by ethnicity.

Ethnicity	Mean value of response				Average of 4 questions
	Question 20^a^	Question 31^b^	Question 32^c^	Question 33^d^	
Caucasian	9.444	7.556	7.667	8.778	8.361
Hispanic	9.325	6.775	7.100	8.650	7.963
Black	9.372	6.302	7.209	7.465	7.587
Asian	9.333	7.000	7.000	9.000	8.083

^a^I trust my doctor.

^b^I would be willing to talk to my doctor about my boyfriend or girlfriend.

^c^I would be willing to talk to my doctor about sex.

^d^I would be comfortable talking to the doctor alone.

**Table 9:** Independent *t* test: questions regarding perception of confidentiality in the doctor-patient relationship. Statistically significant data points have been italicized*.*

Variable	*t* (*df*)	*P* value	Mean difference (SE)	95% CI
Ethnicity	1.515 (81)	.134	0.798 (0.527)	−0.250 to 1.847
Level of parental education	0.606 (94)	.546	0.303 (0.500)	−0.689 to 1.295
Gender	1.851 (95)	.067	0.919 (0.497)	−0.067 to 1.904
Age	−3.781 (95)	*<.001*	−1.747 (0.462)	−2.664 to −0.830
Insurance	2.578 (70)	*.012*	1.477 (0.573)	0.334 to 2.621

**Table 10:** Independent *t* test: questions regarding cultural barriers.

Variable	*t* (*df*)	*P* value	Mean difference (SE)	95% CI
Ethnicity	0.873 (81)	.386	0.375 (0.430)	−0.481 to 1.231
Level of parental education	0.634 (94)	.527	0.232 (0.366)	−0.495 to 0.959
Gender	−0.533 (95)	.595	−0.212 (0.397)	−1.000 to 0.577
Age	−1.802 (95)	.075	−0.691 (0.384)	−1.453 to 0.070
Insurance	1.591 (70)	.116	0.747 (0.469)	−0.189 to 1.683

In section 1 of the survey, 94% of participants acknowledged they had heard the term *electronic health record* before but only 55% answered positively when asked if they knew how EHRs function (Multimedia Appendix 1). In total, 84% of participants expressed interest in viewing their records online and reported a mean score of 8.46 out of 10 when asked if they would like to see the test results online. The majority of participants (77%) used their cell phones as their primary internet access. Only 50% of participants knew that patients aged under 18 years could get treatment for sexual health, mental health, and drug/alcohol abuse without parental consent.

Independent *t* tests performed on *subsection 1: questions regarding perception of confidentiality in the doctor-patient relationship* revealed significant differences for patient age and insurance status but not for ethnicity, level of parental education, or gender. The independent *t* tests for *subsection 2: questions regarding cultural barriers* did not result in significant differences for any of the categories.

## Discussion

The objectives of this study included understanding the (1) perceptions of adolescents with regard to patient-physician relationship, (2) adolescents’ knowledge of EHR systems, and (3) practical concerns of designing an EHR portal that best protects patient confidentiality while helping to facilitate patient-physician communication to improve health outcomes and patient adherence.

### Confidentiality and Cultural Barriers

For the subsection, *perceptions regarding confidentiality in the doctor-patient relationship*, there were statistically significant differences in age (13 to 15 years vs 16 to 18 years) and insurance status (governmental vs private) but not gender, ethnicity, or highest parental education level. These results indicate that the younger age group (aged 13 to 15 years) and those with governmental insurance have more confidence in the confidentiality of their discussions with their physician. These results are concordant with the study by Carlisle et al, which reported older teenagers to be more concerned about confidentiality than younger teenagers [24]. However, if one is to presume that governmental insurance status is correlative to lower socioeconomic status, our results would be contradictory to Ford et al, who reported that adolescents with higher socioeconomic status were more willing to disclose confidential information [6]. Interestingly, there were no statistically significant differences found between gender, ethnicity, or parental education with regard to confidentiality in the physician-patient relationship. These results are discordant with previous studies which demonstrated significant differences between gender and ethnicity [12,24]. The lack of gender results may be due to our limited power owing to the decreased sample size, as well as a male predominance (58 males vs 39 females). With regard to ethnicities, we only compared black with Hispanic patients because of a marginal sample size of other ethnicities; thus, a direct comparison with previous studies is unfortunately inapplicable.

The subsection *questions regarding cultural barriers* had no significant differences for any of the categories including age, ethnicity, gender, or highest level of education. These results are promising in that there were no identifiable cultural barriers to patient’s trust of physicians, which should facilitate improved communication and care given upon implementation of the new patient portal.

For the questions regarding perceptions of confidentiality in the physician-patient relationship, there did not appear to be a strong positive or negative response, with mean results between 4.51 and 5.79 out of 10, indicating that the survey participants may be unsure of what topics are considered to be confidential between them and their physician (Table 1). However, when the results were stratified by race, there seemed to be a marked variation in response to question 22: *My doctor will tell my parents if I am smoking marijuana or drinking alcohol*, ranging from 3.00 and 3.53 for Asian and black participants compared with 4.98 and 7.33 for Hispanic and white participants, respectively. This variation may be due to the differences in how often these individuals see their doctor and the level of communication occurring during those visits, a phenomenon that has been previously documented in the literature [13]. This markedly neutral response to questions regarding confidentiality may also explain some of the results about the knowledge of access to care. Only 50% of survey participants positively indicated that patients aged under 18 years could be treated for depression, drug addiction, and sexually transmitted diseases without their parent’s consent. Furthermore, even fewer (41%) participants were aware that a pregnancy test could be ordered without parental consent (Table 6). These findings demonstrate a possible disconnect in communication and education between providers and the adolescent population. This issue presents as a significant concern as there has been evidence that adolescents who have concerns about confidentiality may forego seeking necessary care [12,23]. If these individuals do forego care, it may lead to significant morbidity, and possibly even mortality, as these sensitive conditions (pregnancy/STIs/substance use/mental health) can have significant short- and long-term side effects.

The findings regarding cultural barriers to care showed that the adolescent population places a great amount of trust in their doctors, averaging 9.33 out of 10 with a minimal score variation between ethnicities. This was offset however by a slightly lower score for questions regarding talking about sex or one’s significant other, with average responses of 6.67 and 7.26, respectively (Tables 5 and 8). These results, although still positive, may be the result of the decreased perception of doctor-patient confidentiality seen in our results, combined with the average age of our study population of 15.5 years. Previous studies have shown that although adolescents do encourage confidentiality, it often comes toward the end of their teenage years [23].

### Understanding and Use of Electronic Health Record Portals

Although 94% of participants have heard of the term *electronic health records*, only 55% knew of their function (Table 2). These findings were supplemented with a noteworthy 84% of patients expressing interest in being able to log in to their EHRs and see their information. These data indicate that the adolescent population is not naïve to the concept of EHRs and are very interested in their content. They, as minors, may simply not have the exposure and formal education about accessing records that an adult with a full EHR access would have. As an essential component of the administration of care, we recommend that physicians educate all developmentally appropriate patients on the content, function, and limitations of their EHR portal. This not only prophylactically addresses potential issues regarding the confidentiality of EHR content but may also further the health literacy and autonomy of adolescent patients [6].

### Design of an Electronic Health Record Portal

The final objective of this study was the practical concerns for the design of an EHR portal. Our findings demonstrate that adolescent patients are very interested in an EHR portal that contains the ability to view test results, current medications, and send messages to their provider (Table 3). These aspects are commonly offered in most adult EHR portals but have been a controversial topic in the pediatric population because of the risk of sensitive content appearing in the portal [16]. Although a proxy access EHR portal would not necessarily prevent these issues, it is expected that this concept would be discussed before parental consent for access, therefore limiting potential future issues. It is also important to note the methods in which EHR portals can be accessed. Previous literature describes limited adoption and access of EHR portals in ethnically diverse populations with low incomes and limited internet access [22]. A total of 77% of survey participants reported that they used their cell phone as their primary tool for accessing the internet. Owing to the relatively low cost and high prevalence of internet-capable mobile phones present in low income populations, the previously described barriers may not be as applicable. Therefore, it is highly recommended that when designing an EHR portal, the system should be easily accessible from a mobile device.

### Limitations

This study has several limitations. The first is that because of the limited sample size, statistical comparisons were only able to be performed between black and Hispanic patients. This somewhat hinders the generalizability of our dataset and limits the identification of ethnically based barriers to EHR portal access. Second, our survey as the first adolescent-directed EHR survey to our knowledge required creation of a de novo and unvalidated question set. Finally, our data collection was primarily performed in a large, urban, academic center, which may not accurately reflect the demographic of patients in the suburban or rural community. In addition, because of the limitations with access to translator services, the survey was only able to be offered in English. This limited the number of patients available to take the survey and may have skewed the dataset away from the large immigrant population present in South Florida.

### Conclusions

The objectives of this study included understanding the (1) perceptions of adolescents with regard to patient-physician relationship, (2) adolescents’ knowledge of EHR systems, and (3) practical concerns of designing an EHR portal that best protects patient confidentiality while helping to facilitate patient-physician communication to improve health outcomes and patient adherence. Our findings demonstrated that although cultural barriers to EHR portal access may be limited, there is a noteworthy deficit in the adolescent population’s understanding of confidentiality and access to care. The results, although preliminary, lend support to the previous studies assessing differences in the understanding of and access to EHR portals in different ethnicities and socioeconomic groups [17-19]. Physicians treating adolescent patients must take an active effort to educate their patients on what topics remain confidential and the availability of resources present in caring for sensitive issues such as substance use, STIs, pregnancy, and mental health. We also recommend that when designing an EHR portal for adolescents, it should be accessible by a mobile device and that it should contain test results, current medications, and the ability to securely message providers. This will allow for easier patient access to their EHR portal, as well as an increase in autonomy, health literacy, and more communication for those who participate. We plan to use the results of this study as the basis for future investigations on EHR portal access in which we may obtain a larger sample size and control group.

## Appendix

Multimedia Appendix 1 42-Item survey distributed to participants.

## Abbreviations

EHR: electronic health record
IRB: Institutional Review Board
STI: sexually transmitted infection
