# Supplementary material for: Understanding of and Barriers to Electronic Health Record Patient Portal Access in a Culturally Diverse Pediatric Population
Source: JMIR Med Inform. 2019 Apr 26;7(2):e11570. doi: 10.2196/11570 (PMC6526688; doi:10.2196/11570)
Supplement: Multimedia Appendix 1 [file medinform_v7i2e11570_app1.pdf]

Pediatric Electronic Medical Record Survey Questions

TO BE FILLED OUT BY PATIENT: ALL SURVEY RESPONSES WILL REMAIN ANONYMOUS

Section 1: What is a medical record and how does it work? *Check only one answer*

Question		
1. I have heard the term "medical record" before.<br>Qualifier: (medical record: the written account of a patient's examination and treatment that includes the patient's medical history and complaints, the physician's physical findings, the results of diagnostic tests and procedures, and medications and therapeutic procedures)	☐ True	☐ False
2. When my parents log into the Hospital Website, they can read about what my doctor and I talked about during the visit.	☐ True	☐ False
3. I'd like to log-in to an Electronic Medical Record so I can see my health information online.	☐ True	☐ False
4. I know how to access my Electronic Medical Record.	☐ True	☐ False
5. Patients can go online to see information about their visit at the doctor.	☐ True	☐ False
6. Only the doctor can see my medical record.	☐ True	☐ False
7. I can see my records on the computer.	☐ True	☐ False
8. When I talk with the doctor, my parent/guardian is always in the room.	☐ True	☐ False

Section 2: Abilities when using a computer *Check only one answer*

9. How good are you at using computers?

☐ Very good ☐ Good ☐ Fairly good ☐ Not good ☐ Prefer not to say

Very Good: can write code, perform graphic design, complete other high level tasks etc.
Good: can use Microsoft office, edit pictures, complete mid-level tasks etc.
Fairly Good: can access social media, send emails, complete standard level tasks etc.
Not Good: have trouble logging in, accessing the internet, and completing basic level tasks etc.

10. Every day I use the internet for: ☐ Less than 1 hour ☐ 1-2 hours ☐ 2-4 hours ☐ More than 4 hours

11. I access the internet mainly through: ☐ Smart phone ☐ Home computer ☐ Public Library ☐ School ☐ Other

12. I use the internet mainly for: **(check all that apply)** ☐ School work ☐ Social Media ☐ Email ☐ Games/Entertainment ☐ Other

13. How would you like your doctor to communicate with you in between visits? **(check all that apply)** ☐ Only at visit ☐ Telephone ☐ Email ☐ Text/instant message ☐ No preference

Section 3: Statement Questions (1 least true, 10 most true) *Circle only one answer*

Statement	Least true									Most true
	1	2	3	4	5	6	7	8	9	10
14. I am comfortable reading in English.	1	2	3	4	5	6	7	8	9	10
15. I am comfortable with my parents seeing my records if it had information about my health.	1	2	3	4	5	6	7	8	9	10
16. I want my record to show what medicines I am taking.	1	2	3	4	5	6	7	8	9	10
17. I want to be able to see my test results online.	1	2	3	4	5	6	7	8	9	10
18. I would be comfortable sending my doctor messages online	1	2	3	4	5	6	7	8	9	10
19. I want to be able to learn about my illness on my online medical record.	1	2	3	4	5	6	7	8	9	10

Section 4: Assessing knowledge of confidentiality *Circle only one answer*

Statement	Least true									Most true
20. I trust my doctor.	1	2	3	4	5	6	7	8	9	10
21. My doctor tells my parents all about our conversations, even those we have in private.	1	2	3	4	5	6	7	8	9	10
22. My doctor will tell my parents if I am smoking marijuana or drinking alcohol.	1	2	3	4	5	6	7	8	9	10
23. My doctor will NOT tell my parents if I am having sex.	1	2	3	4	5	6	7	8	9	10
24. Kids under 18 can get screened for sexually transmitted diseases without their parents' knowledge or required consent.	1	2	3	4	5	6	7	8	9	10

Statement		
25. Sometimes it's hard for me to tell the doctor everything because I'm afraid he or she might judge me.	☐ True	☐ False
26. Parents must be in the room when kids under 18 see the doctor.	☐ True	☐ False
27. Parents must approve for their kids to get any tests.	☐ True	☐ False
28. Patients under 18 can get a pregnancy test at the doctor's office without parent's permission.	☐ True	☐ False
29. Patients under 18 can get treatment for certain conditions like depression, drug addiction and sexual diseases without their parents' knowledge or required consent.	☐ True	☐ False

Section 5: Obtaining care. *Circle only one answer*

	Least true									Most true
30. I will go to the doctor if I am sick.	1	2	3	4	5	6	7	8	9	10
31. I would be willing to talk to my doctor about my boyfriend/girlfriend.	1	2	3	4	5	6	7	8	9	10
32. I would be willing to talk to my doctor about sex.	1	2	3	4	5	6	7	8	9	10
33. I would be comfortable talking to the doctor alone.	1	2	3	4	5	6	7	8	9	10

34. I would be willing to get tested for HIV even if I don't think I have it. ☐ True ☐ False
Qualifier: (HIV or human immunodeficiency virus is a virus that is known to cause damage to the immune system. The virus can only be passed through blood or sexual contact.)

Section 6: Understanding cultural barriers (1 least true, 10 most true) *Circle only one answer*

	Least true									Most true
35. I feel accepted in my family regardless of my sexual preference.	1	2	3	4	5	6	7	8	9	10
36. I am allowed to have a boyfriend/girlfriend.	1	2	3	4	5	6	7	8	9	10
37. If I got checked for a sexually transmitted infection, I would feel embarrassed.	1	2	3	4	5	6	7	8	9	10

38 If I have questions about sex, I have someone to talk to. ☐ True ☐ False

39. My family uses natural medicines instead of going to the doctor. ☐ True ☐ False

40. My family uses both natural medicines & medicine from the doctor. ☐ True ☐ False

41. If I had any questions regarding drugs, sex, or how I am feeling I can talk to: (**check all that apply**)

☐ Parents
☐ Friends
☐ Relatives
☐ Doctor
☐ School professional
☐ Other ____

42. I get check-ups from the doctor: (**check only 1 answer**)

☐ Once a year
☐ Twice year
☐ More than twice a year
☐ Only when I'm sick
